# The Economic Relations of Functional Nervous Diseases

**Published:** 1918-06-08

**Authors:** 


### The Hospital

The Workers' Newspaper of Administrative Medicine and Institutional Life, Administration, National Insurance and Health.

No. 1670, Vol. LXIV. SATURDAY, JUNE 8, 1918. PRICE ONE PENNY

### THE ECONOMIC RELATIONS OF FUNCTIONAL NERVOUS DISEASES.

Functional diseases of the nervous system have always been of interest both to the medical practitioner and to the philosopher, for they are associated not only with questions of practical therapeutics but also with opportunities for speculative inquiries into the constitution of human nature. To the general public they have made no considerable appeal, their incidence being limited and their character failing to invite sympathy. The sufferer, indeed, seems a very close relation of the malingerer, or at least of the egotist, and deserving of stern measures rather than of pity. Thus hysteria, neurasthenia, and suchlike terms often cast a shade of opprobrium on those to whom they are attached, and the victim seems to be a nuisance rather than a patient. The recognition and treatment of these cases prior to the war, though attractive to a certain number of doctors, was hardly a matter of public concern, for the question was a restricted one, and had no large practical application. In short, while the medical profession had a secure knowledge of the reality of these disturbances, there was outside the ranks of the profession a good deal of incredulity, and this of recent years had been emphasised by disputes in connection with the Employers' Liability and kindred Acts of Parliament. Even to the ordinary medical practitioner patients of the variety here in question were apt to be a source rather of irritation than of interest. Monotony, sluggishness, and an obstinacy which comes close to the superfluity of naughtiness are features of the condition, so that even to the initiated there is a touch of unreality in the picture. No wonder, therefore, that the story of functional nervous disease has engaged little public attention, and that even those concerned with individual cases have felt the experience to be a strain upon their patience and forbearance.

Here, as in so many other directions, the war has brought great changes. Under the physical, mental, and emotional stresses of the day the number of the sufferers from functional nervous diseases has very much increased. Among the civil population cases have developed as the obvious consequences of exposure to air raids, but here there have been influences in the opposite direction—such, for example, as the interest of new employments and patriotic duty. Thus the probability is that functional nervous diseases in the general population have been lessened rather than increased by the war. It is in the battlefield and the strain of prolonged campaigns that are to be sought the new and responsible factors, and it is in soldiers tried beyond endurance that the multiplied victims of nerve overstrain have appeared. Hence has arisen the need for special hospitals with favourable opportunities for the study and treatment of these conditions. Of course, this in itself has directed public interest to the question, and has given a sense of reality to complaints which formerly received scanty consideration. Clearly, it is seen that what may be learned from ailing soldiers may in due course be of benefit to ailing civilians. But there is a more immediate and practical side to the question. Soldiers certified as incurable are discharged from the Army, and become State pensioners. This, of course, is only justice, and the country is quite rightly sensitive on such a point. But what if among the men thus discharged there are numbers who are not really incurable, provided only expert diagnosis and appropriate treatment are available? In such circumstances obviously a wrong is done to the individual by condemning him to a life of civilian uselessness, and a wrong is done to the State by inflicting upon it unnecessary expense. Now there are good grounds for believing that at least a third of the unwounded soldiers discharged from the Army are the victims of functional neuroses, and it may be said with confidence that the great majority of these could be completely cured. The figures both of men and money mean a considerable total, and they provide a substantial reason why the public should take a practical interest in functional nervous diseases. The welfare of many individuals is concerned, as are also the interests of the taxpayer. The question is no longer one of mere professional or academic inquiry. Rather it is one of stern moment and financial values.

The essential demands of the situation are evidently accurate diagnosis and appropriate treatment, and both of these have been greatly facilitated by the increase in the number of sufferers. The neurotic character of these diseases has long been known, but recent experiences have shown that the outlook in this direction must be still further enlarged. What are at a superficial glance symptoms by no means suggestive of nerve disturbance have been found again and again to be mere manifestations of functional neuroses, and responsive to the treatment which such neuroses require. Further, and still more important, the treatment of such conditions, if it has not taken on a new form, has taken on a new intensity, with an outcome on the side of time which is truly astonishing. To overcome a functional paraplegia, for example, used to claim months of effort, even if the triumph were ever accomplished. But now we read of such a condition being removed in the course of a few days, or even hours. Hysterical mutism, aphonia, stammering, and tremors come into a similar swift triumph, and thus the whole picture of these conditions is changed. Manifestly it is the duty of the medical profession to impress these possibilities upon the authorities and to urge the establishment of conditions which will make them capable of universal application. The authorities concerned may not be moved by the contemplation of therapeutic triumphs, but they can be counted on when an economic motive is engaged.

